# Genome-Wide Analysis of the Homeobox Gene Family and Identification of Drought-Responsive Members in *Populus trichocarpa*

**DOI:** 10.3390/plants10112284

**Published:** 2021-10-25

**Authors:** Jing Hou, Yan Sun, Lei Wang, Yuanzhong Jiang, Ningning Chen, Shaofei Tong

**Affiliations:** 1 Key Laboratory of Bio-Resource and Eco-Environment of Ministry of Education, College of Life Sciences, Sichuan University, Chengdu 610065, China; Houj237@hotmail.com (J.H.); 21210700102@m.fudan.edu.cn (L.W.); jyz88623@126.com (Y.J.); 2 State Key Laboratory of Grassland Agro-Ecosystems, School of Life Sciences, Lanzhou University, Lanzhou 730000, China; sunyantsf@foxmail.com

**Keywords:** *Homeobox*, phylogenetic analysis, VIGS, *PtrHB3*, *Populus*

## Abstract

*Homeobox* (HB) genes play critical roles in the regulation of plant morphogenesis, growth and development. Here, we identified a total of 156 *PtrHB* genes from the *Populus trichocarpa* genome. According to the topologies and taxonomy of the phylogenetic tree constructed by *Arabidopsis thaliana* HB members, all PtrHB proteins were divided into six subgroups, namely HD-ZIP, ZF-HD, HB-PHD, TALE, WOX and HB-OTHERS. Multiple alignments of conserved homeodomains (HDs) revealed the conserved loci of each subgroup, while gene structure analysis showed similar exon–intron gene structures, and motif analysis indicated the similarity of motif number and pattern in the same subgroup. Promoter analysis indicated that the promoters of *PtrHB* genes contain a series of *cis*-acting regulatory elements involved in responding to various abiotic stresses, indicating that *PtrHB*s had potential functions in these processes. Collinearity analysis revealed that there are 96 pairs of 127 *PtrHB* genes mainly distributing on Chromosomes 1, 2, and 5. We analyzed the spatio-temporal expression patterns of *PtrHB* genes, and the virus-induced gene silencing (VIGS) of *PtrHB3* gene resulted in the compromised tolerance of poplar seedlings to mannitol treatment. The bioinformatics on *PtrHB* family and preliminary exploration of drought-responsive genes can provide support for further study of the family in woody plants, especially in drought-related biological processes. It also provides a direction for developing new varieties of poplar with drought resistance. Overall, our results provided significant information for further functional analysis of *PtrHB* genes in poplar and demonstrated that *PtrHB3* is a dominant gene regulating tolerance to water stress treatment in poplar seedlings.

## 1. Introduction

*Homeobox* gene (HB)-encoded transcription factors contain a highly conserved DNA-binding domain composed of approximately 60 amino acid residues, named the homeodomain (HD). The classical HD structure consists of three alpha-helices and two N-terminal helices. [1]. The HB gene was first identified in *Drosophila melanogaster* in 1983, known as *Antennapedia*; dominant mutations of the gene causes antennae to be replaced by mesothoracic legs [2,3]. Subsequently, a large number of HB genes have been identified in various eukaryotic genomes, including animals, fungi and plants; such transcription factors are highly diversified in their structure and biological function [4], suggesting that these transcription factors may play critical roles in the developmental processes in species across diversiform kingdoms [3,5].

In plants, HB transcription factors have been classified into six groups based on sequence similarity of the functional domains, and with or without other characteristic domains [1]. The latest classification divided the HB family into six groups, namely HD-ZIP (homeodomain leucine zipper), TALE (three-amino-acid-loop-extension), WOX (Wuschel homeobox), HB-PHD (plant homeodomain with a finger domain), ZF-HD (zinc finger with homeodomain) and HB-OTHERS (without characteristic domains) subfamilies [6]. HD-ZIP proteins are found exclusively in plants and consist of another leucine-zipper (ZIP) domain [7], which mediates the protein–protein interaction [5]. ZF-HDs contain two highly conserved zinc-finger-like (PLINC) motifs which participate in protein–protein interactions by mediating homodimerization and heterodimerization [8]. The TALE members contain three additional amino acid residues in the loop that connects the first and second helices of HD [9]. WOX proteins contain additional residues between helix 1 and helix 2, and between helix 2 and helix 3, meanwhile WUS-box motif is composed of eight conserved residues existing in the C-terminal of HD [10]. In addition, the HB-PHD proteins harbor an extra PHD domain upstream of the HD domain [11].

HB transcription factors play critical roles for plant growth, development and response to various biotic and abiotic stresses [12,13,14,15]. For example, two HD-ZIP genes, *OsHOX22* and *OsHOX24*, in rice are induced by different stresses, and the overexpression of *OsHOX24* in *Arabidopsis* enhances sensitivity to abiotic stresses [16]. In *Arabidopsis*, another HD-ZIP gene, *AtHB6*, played an important role in the regulation of ABA signaling by interacting with ABI1 [17]. A TALE gene, *KNAT2*, is expressed in the apical meristem of shoots and is involved in carpel development in *Arabidopsis* [18]. In rice, *OSH1* is required for the maintenance of shoot apical meristem after seed germination [19], and *OSH15* is associated with internode morphogenesis [20]. In soybean, GmSBH1 not only participates in growth and development but also mediates response to humidity and high-temperature stresses [21]. Recently, genome-wide identification and expression pattern analysis of HD-ZIP, TALE and WOX subfamily members have been performed in poplars, with only a few research studies on the biofunctions of poplar HB genes [22,23,24,25]. For example, the expression levels of some TALE genes of poplars responded to salt treatment, indicating their functions associated with salt stress responses [25]. The tissue expression patterns of WOX genes are helpful to identify cambium- and xylem-associated genes, and the overexpression of four WOX genes, namely *WUSa*, *WOX4a*, *WOX5a* and *WOX11/12a* in poplars, influenced adventitious root formation [23].

*Populus* spp. has the characteristics of rapid growth, excellent experimental performance and easy interspecific hybridization and asexual reproduction, it has been selected by scholars as the model plant for forest genomics research. With the publishing of the *Populus trichocarpa* genome [26], the work on gene identification at the whole-genome level is being greatly accelerated. The gene families of MADS-box [27], AP2/ERF [28], NBS [29], WRKY [30], and Heat Shock [31,32] has been performed in *Populus trichocarpa*. Although identification and function analysis of HB genes have been widely carried out in many plant species such as rice, grapes, and carrots [33,34], a comprehensive understanding of the status of HB genes is lacking in *Populus trichocarpa*, and those associated with drought are still unclear. To understand the HB genes in *Populus trichocarpa*, comprehensive analyses of HB genes including the molecular characteristics, gene structure, conserved domain, evolutionary relationship, and expression profile were conducted in our study, and their biological functions in poplar seedlings were further explored via virus-induced gene silencing (VIGS). Our genome-wide results identified all HB genes in the poplar genome, and we first performed an integrated analysis of these members, which contributed to the evolutionary cues of this gene family in poplar and provided the important candidate genes for further functional study. We also demonstrated that VIGS is an efficient tool to study gene functions in poplar seedlings.

## 2. Results

### 2.1. Identification and Classification of HB Genes in P. trichocarpa

**Figure 1:**
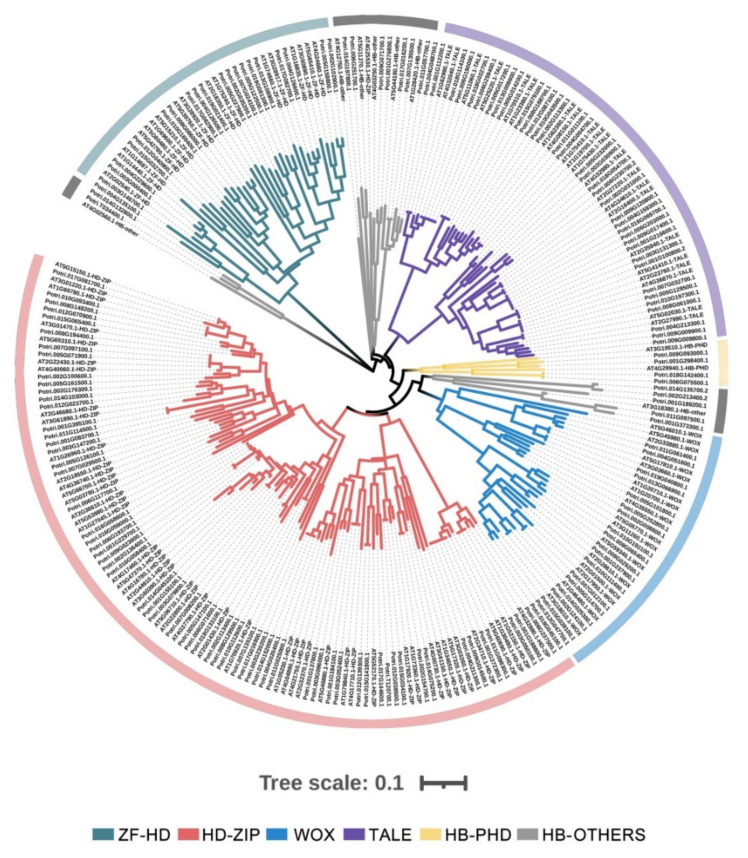
Maximum likelihood tree of the *HB* family from *P. trichocarpa* and *Arabidopsis*. The tree is divided into six subgroups, namely HD-ZIP, ZF-HD, HB-PHD, TALE, WOX and HB-OTHERS, which are indicated with different colors.

Plants evolve multifaceted molecular, physiological and cellular responses throughout their growth, development and stress-resistance process, and various transcription factors, such as HB genes, are often involved in the regulation of these biological processes [7,35,36]. According to the sequence data in the PlantTFDB database, we manually checked these sequences and obtained a total of 156 HB genes in *P. trichocarpa* after removing the alternative splicing forms. In addition, 111 *Arabidopsis* HB genes were also retrieved from its genome. To analyze the phylogenetic relationship of poplar HB genes, we combined all 267 peptide sequences to construct a phylogenetic tree by the maximum likelihood (ML) method (Figure 1). Based on the taxonomy of the *Arabidopsis* HB family [6] and the topological structure of the phylogenetic trees, these HB members were divided into ZF-HD, HD-ZIP, WOX, TALE and HB-PHD, whose members were well clustered; however, the HB-OTHERS members were randomly dispersed throughout the trees but could not be classified into the other five subgroups (Figure 1).

To verify the reliability of the phylogenetic tree constructed with full-length peptide sequences, another phylogenetic tree of conserved HD domains was constructed. The topological structures of these two trees were similar, and the clustering distribution of the ZF-HD, HD-ZIP, WOX, TALE and HB-PHD subfamilies was consistent in both phylogenetic trees, except for much dispersion of the HB-OTHERS members (Figure S1). The largest subfamily of the HB family of *P. trichocarpa* was HD-ZIP, which contained 63 members and accounted for 40.4% (Table S3), which is similar to the proportion of HD-ZIP in rice (48/107), soybean (105/276) and cabbage (71/113). The TALE subfamily contained 35 members, the ZF-HD subfamily contained 21 members, and there were 18 members in the WOX subfamily (Table S3). A total of 15 HB genes belonged to the HB-OTHERS subfamily (Table S3). In addition, the HB-PHD subfamily contained the fewest members (Table S3), which is similar to the results in rice (2), soybean (6) and Chinese cabbage (2) [10,37,38].

**Figure 2:**
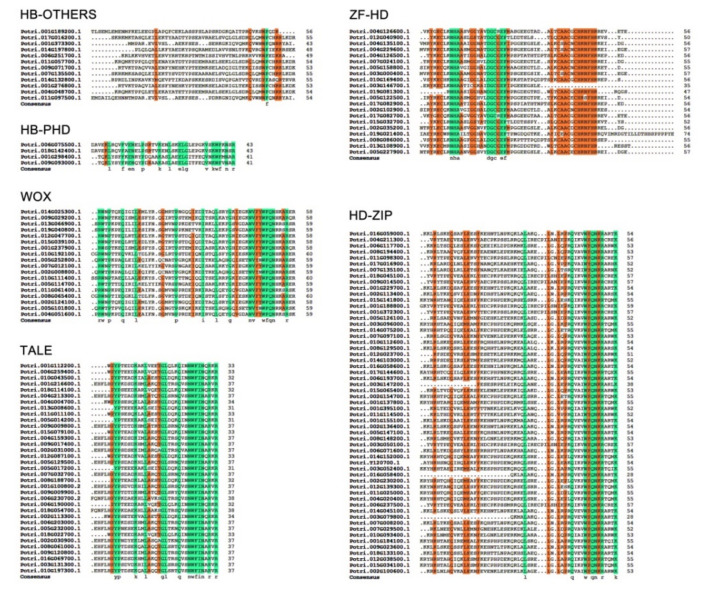
Multiple sequence alignment of conserved HD domains in the ZF-HD, HD-ZIP, WOX, TALE, HB-PHD and HB-OTHERS subfamilies. The colored boxes indicate conserved amino acid residues.

In addition, we examined the sequences of the conserved HD domains from the ZF-HD, HD-ZIP, WOX, TALE, HB-PHD and HB-OTHERS subfamilies. The results indicated that the domains in each subfamily were highly conserved except for HB-OTHERS (Figure 2). The HD domains in the TALE and HB-PHD subfamilies shared the most conserved amino acid residues. For example, the identical amino acid residues of the domains in the TALE subfamily are at Y8, P9, K14, L17, G22, L23, Q7, N30, W31, F32, I33, N34, R36 and R38, accounting for 36.8% of the sequence length of the HD domain. Clearly, the additional three amino acids, P7, Y8 and P9, in the TALE subgroup members are the hallmark characteristics that distinguish TALE from the other subfamilies. The HB-PHD subfamily had the identical amino acid residues at L6, F10, E12, N13, P16, K21, L24, E27, L28, G29, V35, K37, W38, F39, N41 and R43, accounting for 37.2% of the domain (Figure 2). These results indicate that the HD domain sequences of the ZF-HD, HD-ZIP, WOX, TALE and HB-PHD subfamilies of *P. trichocarpa* are conserved, except for HB-OTHERS.

### 2.2. Conserved Motif Analysis

**Figure 3:**
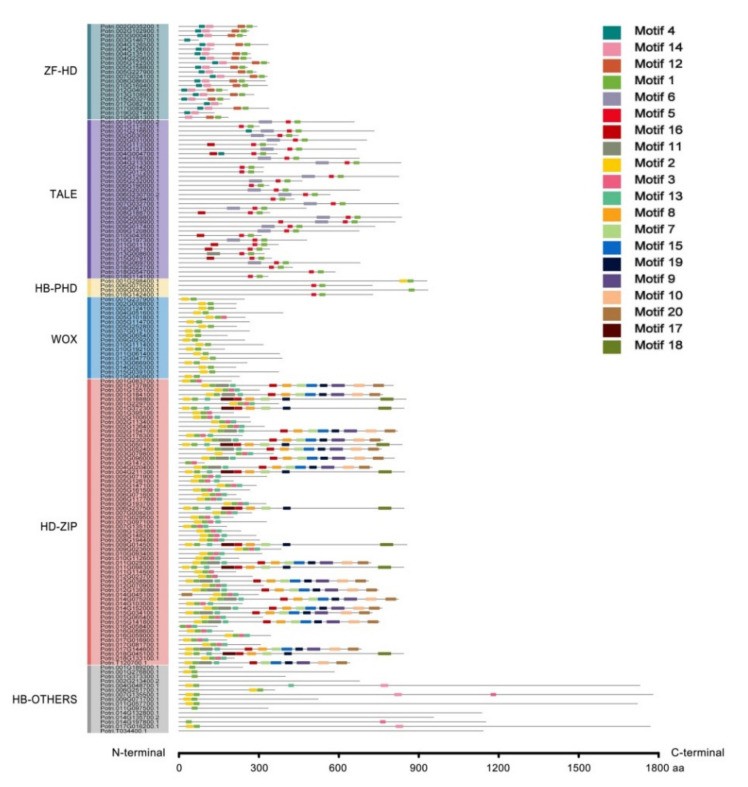
Conserved protein motifs of *PtrHB* members from each subfamily. The motif prediction was performed by the MEME online software. Different color boxes represent different types of motifs.

The conserved motifs on the sequences of 156 *PtrHB* members were analyzed by MEME software. In total, there were 20 motifs with E values less than 1.0 × 10^−200^, and lengths between 20 and 50 amino acids were identified (Table S4). Motifs of the ZF-HD subfamily members were the most conserved; for example, Motifs 4, 14, 12 and 1 were distributed in most members. Motifs 1 and 5 in the TALE family were quite conservative, with each sequence containing both motifs. The N-terminus usually has an additional motif, with a high probability of Motif 6, followed by Motifs 16, 4, or 11 in very few cases. All four members of the HB-PHD were found to have Motif 1 and two to have Motif 5, indicating that HB-PHD is highly similar to the sequences of the TALE members. The WOX members were found to have the most conserved motifs: all sequences contained Motifs 1 and 2, and only Potri005G101800.1 had the additional Motif 5. The HD-ZIP members were found to have long amino acid sequences and a complex motif distribution, which can be classified into four types according to the motif category and distribution order. Type I has five proteins; the distribution order from the N-terminal to the C-terminal is Motifs 2-1-3. The C-terminal motif distribution of 2-1-3-13 was classified as Type II with 32 members. Type III is Motifs 2-1-13-17-16-8-7-19-18 with eight proteins. The Type IV sequence information is Motifs 2-1-11-13-16-8-7-15-19-9-10-20 with 16 members. The motif distribution of HB-OTHERS could be roughly divided into three types, namely Motif 1, Motifs 2-1 and Motifs 2-1-14, and interestingly no conserved motifs were found in another four proteins (Figure 3). In conclusion, the distribution of conserved motifs in each subfamily was similar.

Interestingly, all HB members contain Motif 1, which occupies the largest advantage among all 20 motifs, with a high proportion having Motifs 2 and 5, but the four HB-OTHER members have no conserved motif (Figure 3). Therefore, Motifs 1, 2 and 5 can be considered to be highly conserved and widely distributed in HB proteins. Some motifs were specific: for example, Motif 12 appeared only in ZF-HD proteins, Motifs 4 and 6 mainly appeared in TALE members, while Motifs 7, 8, 9, 10, 11, 18, 19 and 20 appeared only in the HD-ZIP subfamily (Figure 3), suggesting that these motifs may be related to the specific functions of the corresponding subfamily members.

### 2.3. Gene Structure Analysis

**Figure 4:**
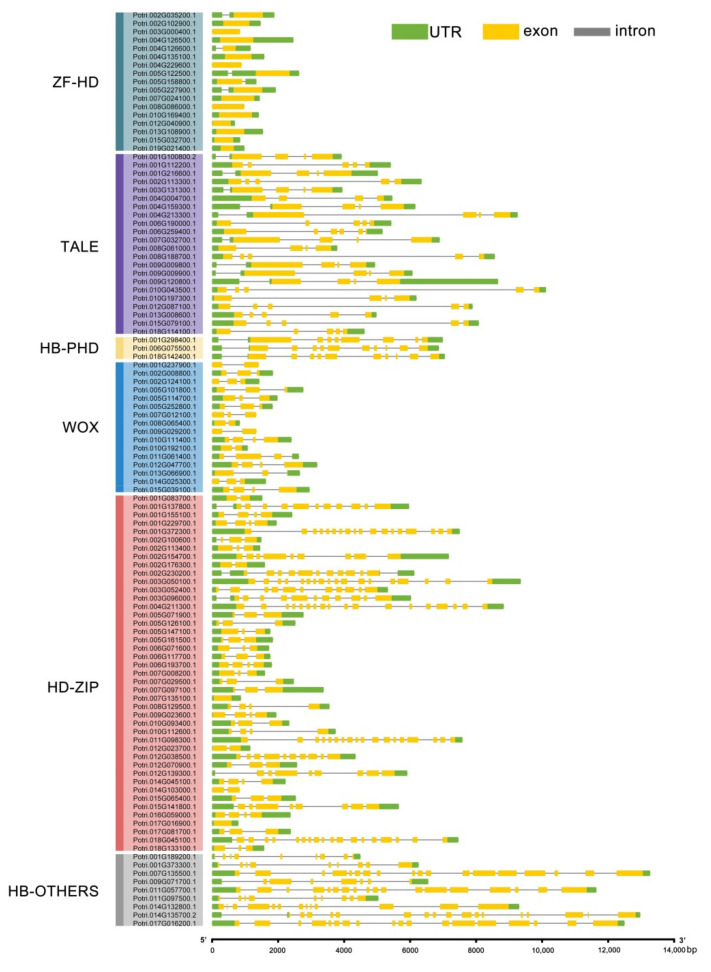
Structural analysis of *PtrHB* genes in P. trichocarpa. Introns, exons and UTR regions are represented by gray lines, yellow boxes and green boxes, respectively.

The gene structures of *PtrHB* genes were constructed by TB Tools software, indicating the exon number and relative locations of genes. The genes in the same subfamily showed a similar structure pattern, and almost all genes have UTR regions in both terminals (Figure 4). The number of introns was similar in the genes from the same subfamily. The genes from the ZF-HD, TALE, WOX and HB-PHD subfamilies have few introns and the locations are relatively constant. The ZF-HD genes have either no introns or only one intron, most genes of the TALE subfamily contain three or four introns, three HB-PHD genes have nine introns and the WOX genes have no more than three introns. The genes from the HD-ZIP and HB-OTHERS subfamilies have a large number of introns. Many HB-OTHERS subfamily genes contain 17 introns, and some HD-ZIP genes contain 18 introns (Figure 4). Therefore, the gene structures of the same subfamily genes are similar, but the length and location of these introns in every subfamily are very different.

### 2.4. The Prediction of Cis-Acting Elements

**Figure 5:**
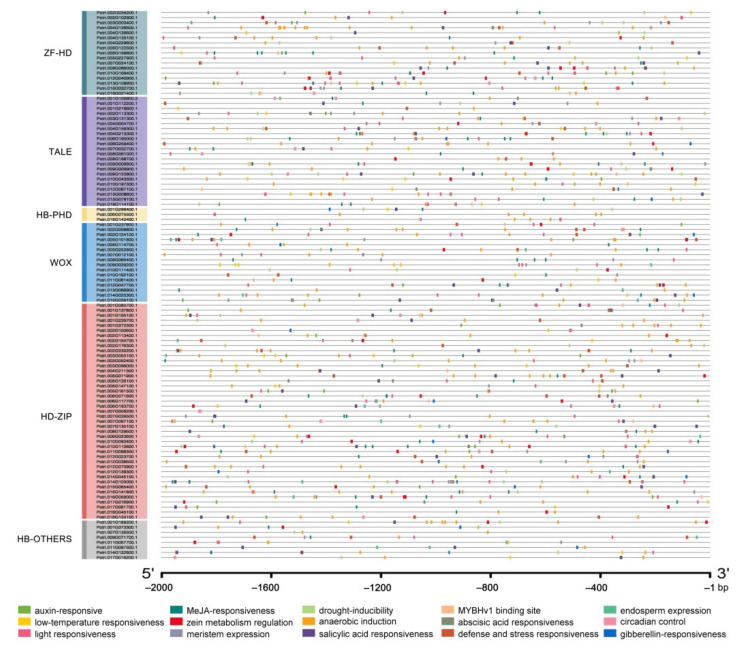
Distribution of major cis-acting elements in the promoters of the poplar PtrHB genes. Putative cis-acting elements, including auxin-responsive, MeJA-responsiveness, drought-inducibility, MYBHv1 binding site, endosperm expression, low-temperature responsiveness, zein metabolism regulation, anaerobic induction, abscisic acid responsiveness, circadian control, light responsiveness, meristem expression, salicylic acid responsiveness, defense and stress responsiveness and gibberellin responsiveness, are visualized by different color boxes.

PlantCARE online software was used to predict *cis*-acting elements in the promoter region of 2000 bp upstream of the starting codon of all *PtrHB* genes. A total of 18,271 elements were obtained, but after excluding core promoter elements such as CAAT-box and TATA-box, as well as elements with incomplete annotation information, 1406 *cis*-acting elements belonging to 15 typical types were obtained. Distinguishing from the conserved protein motifs, both the number and type of the *cis*-acting element had no specificity in each of the subfamilies (Figure 5). We noted that the *cis*-acting elements in *HB* promoters are mainly involved in defense and stress responses, such as anaerobic, chilling and drought-stress responses. The anaerobic element had the most number, followed by the light-response element; these two types of elements are in most genes promoters, meanwhile these genes contain several identical elements. Some *PtrHB* genes are involved in regulating plant responses to hormones including gibberellin (GA), abscisic acid (ABA), salicylic acid (SA) and auxin (Table S6), consistent with the previous reported [17,39,40]. The ABA-response element is most widely distributed in almost all the promoters of *PtrHB* genes (Table S6). Promoter analysis also suggested that *PtrHB* genes may be associated with the circadian clock, protein metabolism, cell differentiation and morphogenesis regulation.

### 2.5. Chromosomal Localization and Synteny Analysis

**Figure 6:**
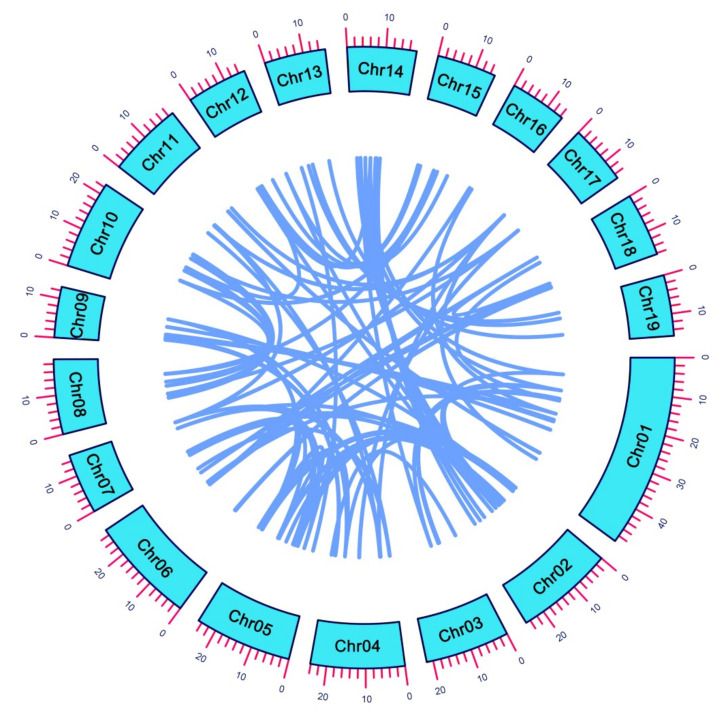
Chromosomal location and synteny analysis of poplar PtrHB genes. The Circos diagram indicates the chromosomal location and collinearity of the PtrHB gene in poplars.

*PtrHB* genes were located on all 19 chromosomes according to the annotation of the *P. trichocarpa* genome (Table S5). The *PtrHB* genes were more abundant on Chromosomes (Chrs) 1, 2 and 5, carrying 12, 13 and 12 genes, respectively, and accounted for about one third of the total number of *PtrHB* genes, indicating that the three chromosomes were evolutional hot spots in *PtrHB* genes. Five chromosomes (Chrs 6, 7, 9, 10 and 14) carried seven *PtrHB* genes, four chromosomes (Chrs 8, 11, 12 and 15) carried six, three chromosomes (Chrs 3, 13 and 17) carried four and Chrs 16, 18 and 19 contained 2, 3 and 5 *PtrHB* genes, respectively (Table S5). The MCScanX software was used to obtain the synteny relationship of the *PtrHB* genes. We obtained the synteny relationships of a total of 96 pairs of 127 HB genes, and Circos software was used to visualize the results (Figure 6).

### 2.6. Expression Patterns of PtrHB Genes

**Figure 7:**
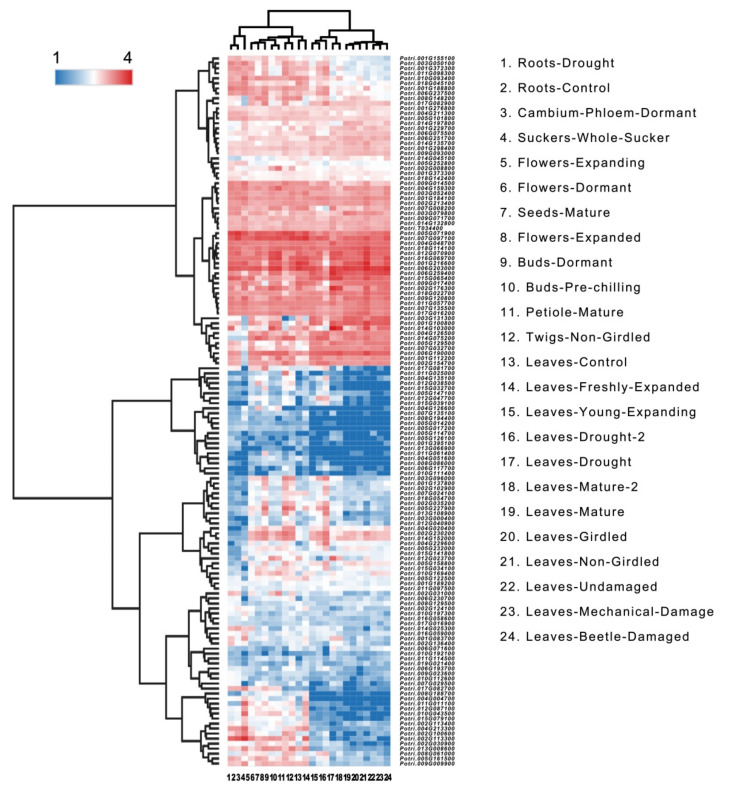
Heatmap of the expression pattern of PtrHB genes in the roots, flowers, seeds, buds and leaves of Populus at different developmental stages and under different environmental conditions.

The expression patterns of genes provide information on their biofunctions [36]. To clarify the expression patterns of HB genes in *P. trichocarpa*, we analyzed 24 independent transcriptome data to construct a heatmap of gene expressions and cluster analysis (Table S1). According to the tissue expression patterns of *PtrHB* genes, the heatmap was significantly divided into two branches, and majority genes expressed in leaves were significantly lower than those in roots, buds and seeds. The expression patterns of the HD-ZIP and TALE genes were very different, suggesting their functional differences in poplars. Most HB-OTHERS and almost all HB-PHD genes showed high expression levels (Figure 7). In addition, most genes from the ZF-HD and WOX subfamilies have very low expression levels (Figure 7). In addition, we found that drought stress induced some *PtrHB* genes in both leaves and roots through comparing Sample 1 (roots-drought) with 2 (roots-control) and comparing Sample 13 (Leaves-Control) with 16 (Leaves-Drought-2) and 17 (Leaves-Drought). The transcription levels of four genes, namely *PtrHB1* (*Potri.006G203000.1*), *PtrHB3 (Potri.006G259400.1*), *PtrHB5* (*Potri.015G065400.1*) and *PtrHB12* (*Potri.002G176300.1*), were significantly up-regulated (Figure 7). Therefore, we further investigated the physiological functions of these four genes in response to drought-associated stresses.

### 2.7. The Spatio-Temporal Expression Patterns of PtrHB1, PtrHB3, PtrHB5 and PtrHB12

**Figure 8:**
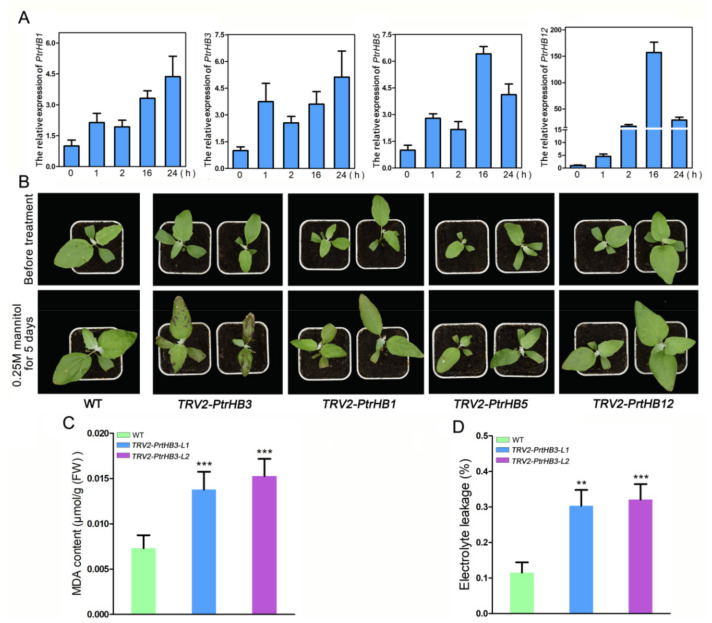
Expression patterns of *PtrHB1*, *PtrHB3*, *PtrHB5* and *PtrHB12* responsive to mannitol treatment, and the phenotypes of their silenced lines under mannitol treatment: (**A**) expressions of *PtrHB1*, *PtrHB3*, *PtrHB5* and *PtrHB12* were up-regulated by mannitol treatment; (**B**) phenotypes of *PtrHB1*, *PtrHB3*, *PtrHB5* and *PtrHB12* silenced lines under mannitol treatment; (**C**,**D**) MDA content and electrolyte leakage of WT and silenced lines after mannitol treatment (**, *p* < 0.05, ***, *p* < 0.001).

To verify the expression patterns of *PtrHB1*, *PtrHB3*, *PtrHB5* and *PtrHB12* in poplars when dehydration occurs, mannitol treatment mimicking drought stress was performed [37,38]. The expression levels of these four genes were significantly up-regulated after treatment with 0.25 M mannitol (Figure 8A). The expression levels of *PtrHB1* and *PtrHB3* increased gradually with time and reached the maximum expression level at 24 h after treatment, which was about 4.5 times that before treatment (Figure 8A). The expression levels of *PtrHB5* and *PtrHB12* reached the peak at 16 h after treatment, and then decreased at 24 h, results that were still much higher than those before treatment (Figure 8A). Interestingly, *PtrHB12* was up-regulated about 150 times by the mannitol treatment, due to the low basal expression level under normal conditions (Figure 8A). These qPCR results supported the transcriptome data and suggested that *PtrHB1*, *PtrHB3*, *PtrHB5* and *PtrHB12* might be involved in the drought-stress response in poplar.

We explored the tissue expression patterns of *PtrHB1*, *PtrHB3*, *PtrHB5* and *PtrHB12* in poplars. The results showed these four genes have similar tissue expression patterns; they prefer expressing in petioles and old leaves to roots and young leaves (Figure S2). The expression levels of *PtrHB1*, *PtrHB5* and *PtrHB12* were similar in petioles and old leaves, while *PtrHB3* was nearly three times more in old leaves than that in petioles (Figure S2). The mechanisms of enhanced tolerance to drought stress are related to leaf traits, which are often used as criteria to evaluate plants’ drought-stress tolerance [39]. Therefore, the high expression of *PtrHB1*, *PtrHB3*, *PtrHB5* and *PtrHB12* in old leaves suggested that they may be involved in the regulation of drought-stress-related pathways in leaves.

### 2.8. Silencing PtrHB3 Results in Decreased Tolerance to Mannitol Treatment

Virus-induced gene silencing (VIGS) is a technique to inhibit the translation of coding genes and has been widely used to study the biological functions of plant genes, avoiding the dilemma of traditional transgenes requiring plant regeneration [40,41,42]. Phytoene desaturase (PDS) is highly conserved in the plant kingdom, and it has been widely used as a marker gene for detecting VIGS efficiency; the silencing of *PDS* results in white leaves caused by photobleaching [43,44]. To demonstrate the efficiency of VIGS in poplar seedling, we selected the poplar seedlings after 2 weeks of germination and their leaves were transfected with *Agrobacterium* containing both TRV2-PDS and TRV1 vectors. After two weeks of infection, the photobleached leaves were observed, demonstrating that the system could successfully induce endogenous gene silencing in poplar (Figure S3). When studying the biological functions of target genes in woody plants, genetic transformation is extremely difficult and requires too much time, which seriously restricts the research progress. The successful application of VIGS in poplars provided an effective method and tool for later researchers to quickly determine the gene function in woody plants. 

To identify the biofunctions of *PtrHB1*, *PtrHB3*, *PtrHB5* and *PtrHB12* in the tolerance to drought stress, we co-infected the poplar seedlings with the *Agrobacterium* containing TRV2-PtrHB1, TRV2-PtrHB3, TRV2-PtrHB5 and TRV2-PtrHB12 constructs combined with TRV1, respectively. The infection efficiency of the virus was identified by detecting the coding sequence of the moving proteins expressed by the TRV1 plasmid (Figure S4). The silencing efficiency of the four genes in the seedlings was detected by qPCR (Figure S5).

Compared with the controls, the silenced lines of *PtrHB1*, *PtrHB5* and *PtrHB12* showed no obvious phenotype differences after 5 days of mannitol treatment. However, silencing *PtrHB3* showed a hypersensitive phenotype to this osmotic stress. We noted that the leaves of silenced seedlings were severely distorted and blackened and many spotted necroses appeared (Figure 8B). In addition, the malondialdehyde (MDA) content in *PtrHB3* silenced seedlings (MDA content is about 0.015 μmol/g, fresh material) was much higher than that of WT (MDA content is about 0.0073 μmol/g, fresh material) (Figure 8C). The electrolyte leakage (EL) in *PtrHB3* silenced lines (EL ratio is about 0.3) was more than twice that of WT (EL ratio is about 0.1) (Figure 8D). These results demonstrated that *PtrHB3* positively regulated the tolerance of poplar seedlings to drought stress. In the future, silencing or overexpressing *PtrHB3* in poplar by genetic transformation, the regulatory network and biological processes involved in *PtrHB3* can be explore by transcriptome and metabolome, and decoded the precise genetic function.

## 3. Discussion

Homeobox proteins are found in invertebrates, fungi, vertebrates and plants and contain a conserved DNA-binding domain known as the HD [45]. HB genes play a vital function in plant growth and development, as well as in stress responses [26,41]. HB genes exist in large numbers of land plants and expand during angiosperm evolution [46,47]. For example, the *Arabidopsis* genome has experienced four different large-scale duplication events, and the large number of HB genes in many plants are likely as the result of genome duplication [48]. It is necessary to identify all the HB members at a whole-genome level in *Poplar. trichocarpa*.

To investigate the relationships of HB genes in poplar, we investigated 156 poplar *PtrHB* genes, which contain many more members than rice (107), cabbage (113) and *Arabidopsis* (111) [49,50]. The HB family expansion in poplar may be related to genome duplication. *PtrHB* genes distribute unevenly on all 19 chromosomes according to the annotation of the *P. trichocarpa* genome, and Chrs 1, 2 and 5 accounted for about one third of the total number of *PtrHB* genes (Table S5, Figure 6); therefore, there were evolutionary hot spots of HB genes in these three chromosomes. Similar results were also found in other species: for instance, there were 13 ZF-HD genes distributed on 6 chromosomes of the cucumber genome, while Chr 5 carried the most ZF-HD genes [51]. In tomato, 22 ZF-HD genes were distributed on 12 chromosomes, but the distribution was extremely uneven [48].

According to the classification of the HB genes family in *Arabidopsis* and the topological structure of the phylogenetic tree, the HB genes of *P. trichocarpa* were divided into six subfamilies, namely HD-ZIP, ZF-HD, HB-PHD, TALE, WOX and HB-OTHERS (Figure 1). Another phylogenetic tree was constructed with the conserved HD domain, and the sequence alignment of these domains supported this phylogenetic relationship and taxonomy (Figure 1 and Figure S1). This also indicates that the structural conserved properties within gene families provide important evidence for the study of genomic evolutionary relationships [52]; therefore, we systematically analyzed the distribution of the exon and intron structure of the HB genes in poplar. The structure of each gene within each subfamily is relatively similar (Figure 4), which further supported our taxonomy of *PtrHB* genes (Figure 1, Figure 2 and Figure S1). Among the six subfamilies, the HD-ZIP subfamily is the largest, containing 63 members, and the smallest is the HB-PHD subfamily with only 4 members, which is consistent with the results in other plants, indicating that the proportion of subfamilies in different plants is relatively consistent [53,54,55].

Multiple sequence alignment of the HD domains revealed conserved amino acid residues and specific structures among members of each subfamily (Figure 2), such as the additional PYP amino acid domain in the TALE family. PYP connects the region between the first and second helixes of TALE, and all TALE members share three homologous domains with PYP, except for TALE29, in poplar [25,56]. These results reflected the conservatism of the *PtrHB* family and the differences among the different subfamilies in poplar. Analyzing the conserved motifs of proteins revealed that the HB superfamily members had partially conserved motifs, and the genes of each subfamily had similar motif species and distribution patterns (Figure 3). The protein motifs are important to their functions: for example, the leucine zipper is responsible for the interactions between proteins. We found that the leucine zipper was mainly in the HD-ZIP proteins of poplars (Figure 3 and Table S3), indicating that these members function in the form of homodimers or heterodimers. The conserved of the motifs does not fully account for functional similarity. In the future, the gene function judged by conserved motifs need to be verified by genetic transformation in poplars. 

Transcription factors such as *trans*-acting factors are required for binding to *cis*-acting elements in gene promoters or enhancers to regulate gene expressions in various biological processes [50]. We analyzed *cis*-acting elements in the 2 kb promoter region of *PtrHB* genes and found that these promoters contained a variety of *cis*-acting elements involved in hormone and abiotic stress responses, including methyl jasmonate (MeJA)-response elements, light-response elements, ABA-response elements and anaerobic-induction-response elements (Figure 5). These results were similar in Chinese cabbage, with most HB gene promoters containing *cis*-acting elements related to light response, hormone response and stress response [50]. Promoter analysis indicated that *PtrHB* genes may be involved in the regulation of hormone response and stress response in poplars. To verify the response of *PtrHB* to hormone and abiotic stress, exogenous hormone or stress treatment were applied to poplar, and then the transcription level of *PtrHB* genes were detected to judge the response, in further research.

HB genes participate in various growth and development processes and regulate responses to stress responses in many plant species [57,58]. In *Brassica rapa*, the expression pattern of *BraHB*s showed dynamic changes and responses to various stresses [50]. In rice, at least 37 HB genes were significantly differentially expressed more than twice under various abiotic stress conditions [49]. In poplar, we found the expression levels of most *PtrHB*s were lower in leaves than those in roots, buds and seeds regardless of stress condition or developmental condition (Figure 7). Moreover, we found that drought stress could induce *PtrHB1*, *PtrHB3*, *PtrHB5* and *PtrHB12* in both leaves and roots (Figure 7), which had been verified by qPCR (Figure 8), suggesting their potential functions in response to drought stress. We investigated the functions of these four genes through knocking down their expression levels in *P. alba* by VIGS and demonstrated the expression of *PtrHB3* is indispensable to drought tolerance in poplar seedlings. This is the first report to show that VIGS is efficient in poplar seedlings after germination. However, the limitation of the VIGS technique is that the phenotypes of most lines are not heritable, so biological function of genes cannot be further studied in progeny. In addition, the efficiency of gene silencing induced by VIGS is not very efficient. In poplars, VIGS can help researchers quickly determine the biological function of genes so that they can select target genes for further study. Fortunately, our results share important information on the functional identification of poplar *PtrHB* genes and provide the phenotype cues of *PtrHB3* to further study the molecular regulatory mechanisms in poplars.

## 4. Materials and Methods

### 4.1. Genome Information

*P. trichocarpa* genome and annotation information were downloaded from the PHYTOZOME (v4.1) database (https://phytozome-next.jgi.doe.gov/; accessed on 12 April 2020). The genetic information of *A. thaliana* and *P. trichocarpa* is derived from the plant transcription factor number database (http://planttfdb.gao-lab.org/index.php; accessed on 15 April 2020).

### 4.2. HB Gene Family Identification and Phylogenetic Tree Construct

MEGA was used to construct the phylogenetic tree and the multiple sequence alignment of the HB family proteins in *P. trichocarpa* and *Arabidopsis* using the maximum likelihood tree (ML) with 1000 bootstrap replications. In addition, the same method was used to construct a phylogenetic tree of the HD domains of *P. trichocarpa*. The classification of the poplar HB superfamily was based on the taxonomy of *A. thaliana.*

### 4.3. Sequence Alignment

The HD domain sequences of the poplar HB proteins were downloaded from the PlantTFDB website (http://planttfdb.gao-lab.org/; accessed on 21 April 2020), Clustal W was used for multiple sequence alignment in MEGA and the conservation degree (100% or >75%) was indicated by the DNAMAN software.

### 4.4. Analysis of Motif and Gene Structure

The online tool of MEME (https://meme-suite.org/meme/; accessed on 22 April 2020) was used to analyze the motifs of PtrHB proteins: the length range of the motifs was from 6 to 50 amino acid residues and the total number of motifs was no more than 20. The exon–intron gene structure was mapped in TB Tools according to the downloaded genome annotation information.

### 4.5. Synteny Analysis of PtrHB Genes

MCScanX was used to obtain the synteny data of all the genes of *P. trichocarpa*. The correlation information of the HB genes was selected, and the correlation color was added to make the graph using the Circos software [59].

### 4.6. Promoter Analysis of cis-Acting Elements

The 2 kp length of the promoter regions of the *PtrHB* genes upstream of the initiation codon was retrieved from the genome annotation file of *P. trichocarpa* using TB Tools. The *cis*-acting elements in the promoter sequences were predicted using the online site PlantCARE (http://bioinformatics.psb.ugent.be/webtools/plantcare/html/; accessed on 25 April 2020). In the prediction results, the conservative landmark elements of the promoters of eukaryotic protein-encoding genes, such as TATA-box and CAAT-box, were deleted. We selected the *cis*-acting elements with complete annotations, with the top 15 occurrence frequencies, for visual analysis by TB Tools.

### 4.7. Gene Expression Analysis

All the expression data of *P. trichocarpa* HB genes were downloaded from the PopGenIE database (https://popgenie.org/start; accessed on 26 April 2020). Tophat and Cufflink software programs were used to analyze the gene differential expressions in the transcriptomes. The log2 values (TPM) were applied to construct the heatmap to display the gene expression and the cluster analysis by R.

### 4.8. Plant Material and Growth Condition

The poplar seeds (*P. alba*) were sterilized and directly germinated on MS medium for 7 days, and then we selected and transplanted the well-developed seedlings into nutrient soil. The seedlings were cultured in the greenhouse at 25 °C under light/darkness of 16/8 h with a light intensity of 4500 lux for the subsequent experiments, as previously reported [38].

### 4.9. The qPCR Analysis

The RNA was extracted from fresh poplar leaves using the BIOFIT plant RNA extraction kit (V1.5; Biofit Biotechnologies, Chengdu, China), and then the concentration and quality of the total RNA was detected by a NanoDrop 2000 spectrophotometer (Thermo Fisher Scientific, Waltham, MA, USA). According to the protocol of the HifairIII1st Strand cDNA Synthesis Super Mix (YEASEN, Shanghai, China) reagent kit, 2 μg of total RNA (after digestion of genomic DNA contained in total RNA) was reverse transcribed into cDNA for each sample. Real-Time EasyTM-SYBR GreenI (FORE GENE Bio Inc., Chengdu, China) was used to analyze gene expression by qPCR. The C1000 Touch Thermal Cycler (BIO-RAD, Hercules, CA, USA) was used for qPCR, and the parameters were 95 °C for 5 min, followed by 40 cycles of 95 °C for 5 s and 60 °C for 35 s. The DNA sequence of *BUQ* was used as an internal control. The relative expression levels of the genes were calculated according to the previously reported method [60]. The gene-specific primers used in the experiment are listed in Table S2.

### 4.10. Gene Cloning and Plasmid Construct

The gene fragments of *PtrHB1* (*Potri.006G203000.1*), *PtrHB3* (*Potri.006G259400.1*), *PtrHB5* (*Potri.015G065400.1*) and *PtrHB12* (*Potri.002G176300.1*) were amplified from the cDNA with Phanta Max Super-Fidelity DNA Polymerase (Vazyme, Nanjing, China) according to the instructions. The VIGS-TRV system was used in this study as previously reported, and the PCR products were digested and ligated into TRV2 digested by XhoI and EcoRI restriction enzymes [43]. The recombinant plasmid was transformed into *Agrobacterium* strain GV3101 by the freezing–thawing method.

### 4.11. Agrobacterium Infiltration

For the VIGS experiment, the positive strain GV3101 containing TRV1 or TRV2 plasmid was cultured overnight in the YEP medium containing three antibiotics (50 mg/L rifampicin, 50 mg/L gentamicin and 50 mg/L kanamycin) in conditions of 28 °C and 180 rpm. The next day, each Agrobacterium culture was inoculated into the new YEP medium to cultivate until the O.D._600_ was 1.0, and then the Agrobacterium cells were harvested for suspension in the MMA buffer (10 mM MES, 10 mM MgCl2, 200 μM acetosyringone, pH 5.7) with O.D._600_ = 0.8. Before infection, the Agrobacterium suspensions were incubated in conditions of 28 °C and 150 rpm for 4 h, and then, the Agrobacterium cultures containing TRV1 and TRV2 were mixed in a 1:1 ratio. The 7-day-old poplar seedlings were submerged in the Agrobacterium mixture and vacuumed at 0.08 MPa for 15 min. Finally, the seedlings were transplanted into soil for normal growth, and after two weeks, the seedlings were treated with 0.25 M mannitol solution.

### 4.12. Measurement of MDA and Conductivity

A thiobarbituric acid (TBA) method was used for the quantification of MDA content in poplar seedlings [61]. Electrolyte leakage (EL) was measured according to the mature system previously reported [62].

### 4.13. Statistical Analysis

The experimental data were subjected to SPSS (SPSS Statistics 17.0, 2008) for statistical tests and analyses. Tests of outliers and normality were performed prior to statistical analysis. Unless otherwise stated, *p* < 0.05 was considered to be significant.

## Data Availability

The *P. trichocarpa* genome and annotation information were downloaded from the PHYTOZOME (v4.1) database (https://phytozome.jgi.doe.gov/pz/portal.html#?tdsourcetag=s_pcqq_aiomsg; accessed on 12 April 2020). The genetic information of *A. thaliana* and *P. trichocarpa* was derived from the plant transcription factor number database (http://planttfdb.gao-lab.org/index.php; accessed on 15 April 2020). The HD domain sequences of the poplar HB proteins were downloaded from the PlantTFDB website (http://planttfdb.gao-lab.org/; accessed on 21 April 2020). The abundance of *PtrHB* gene expressions used in the heatmap can be retrieved in Table S1.

## Supplementary Materials

The following are available online at https://www.mdpi.com/2223-7747/10/11/2284/s1. Figure S1: Phylogenetic analysis of HD-conserved domain of *PtrHB* genes in poplar; Figure S2: Expression levels of *PtrHB1*, *PtrHB3*, *PtrHB5* and *PtrHB12* in poplar roots (R), stems (S), petioles (P), old leaves (OL) and young leaves (YL); Figure S3: *PDS* silencing phenotype induced by virus-induced gene silencing in Populus. Bleached leaves indicated that *PDS* had been successfully silenced; Figure S4: Identification of TRV infected and spread in the poplar seedlings by detecting the moving protein coding sequence of TRV by PCR; Figure S5: Expression levels of *PtrHB1*, *PtrHB3*, *PtrHB5* and *PtrHB12* in silenced seedlings were detected by qPCR; Table S1: Expression levels of *PtrHB* genes in *P. trichocarpa*; Table S2: Primers used in the research; Table S3: Subfamily proportion of *PtrHB* gene in *P. trichocarpa*: Table S4: Motifs associated with PtrHB proteins in *P. trichocarpa*; Table S5: Chromosomal location of *PtrHB* genes associated with the synteny in *P. trichocarpa*; Table S6: Main *cis*-regulatory elements located in *PrtHB* promoters.
