# Supplementary figures and images for: Genome-Wide Analysis of the Homeobox Gene Family and Identification of Drought-Responsive Members in Populus trichocarpa
Source: Plants (Basel). 2021 Oct 25;10(11):2284. doi: 10.3390/plants10112284 (PMC8653966; doi:10.3390/plants10112284)

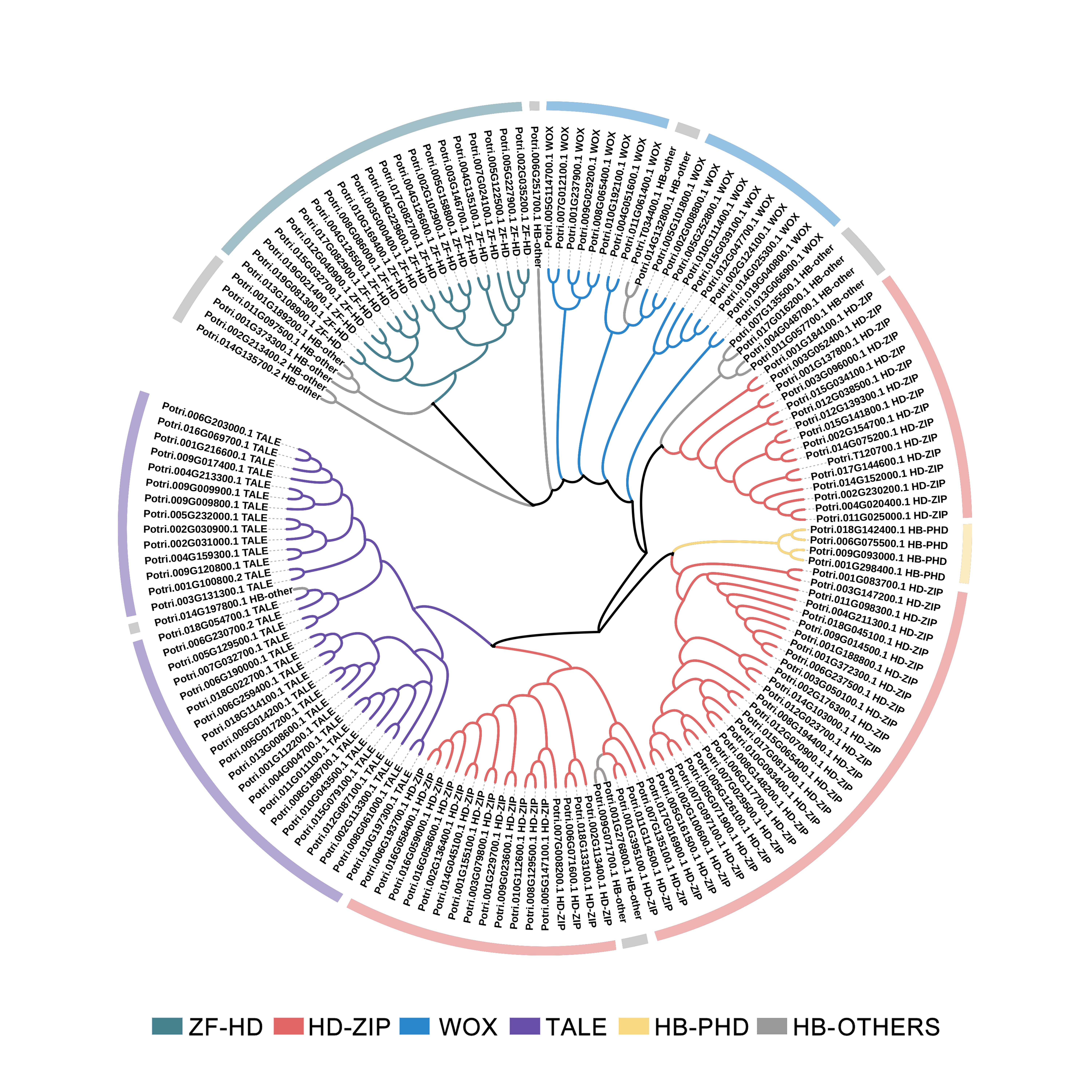

Supplement: Supplementary file 1 [file plants-10-02284-s001.zip › S1.jpg]

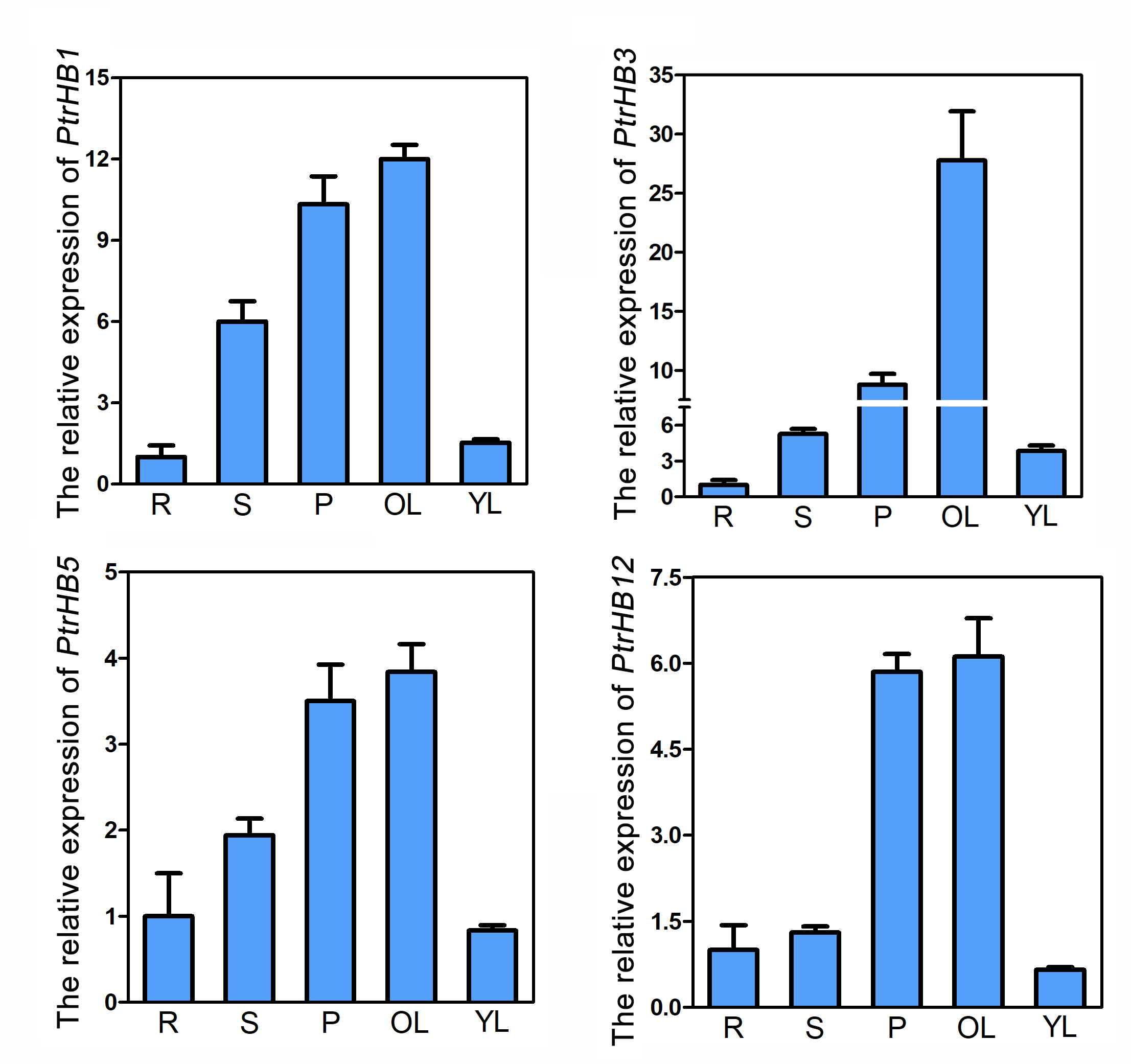

Supplement: Supplementary file 1 [file plants-10-02284-s001.zip › S2.tif]

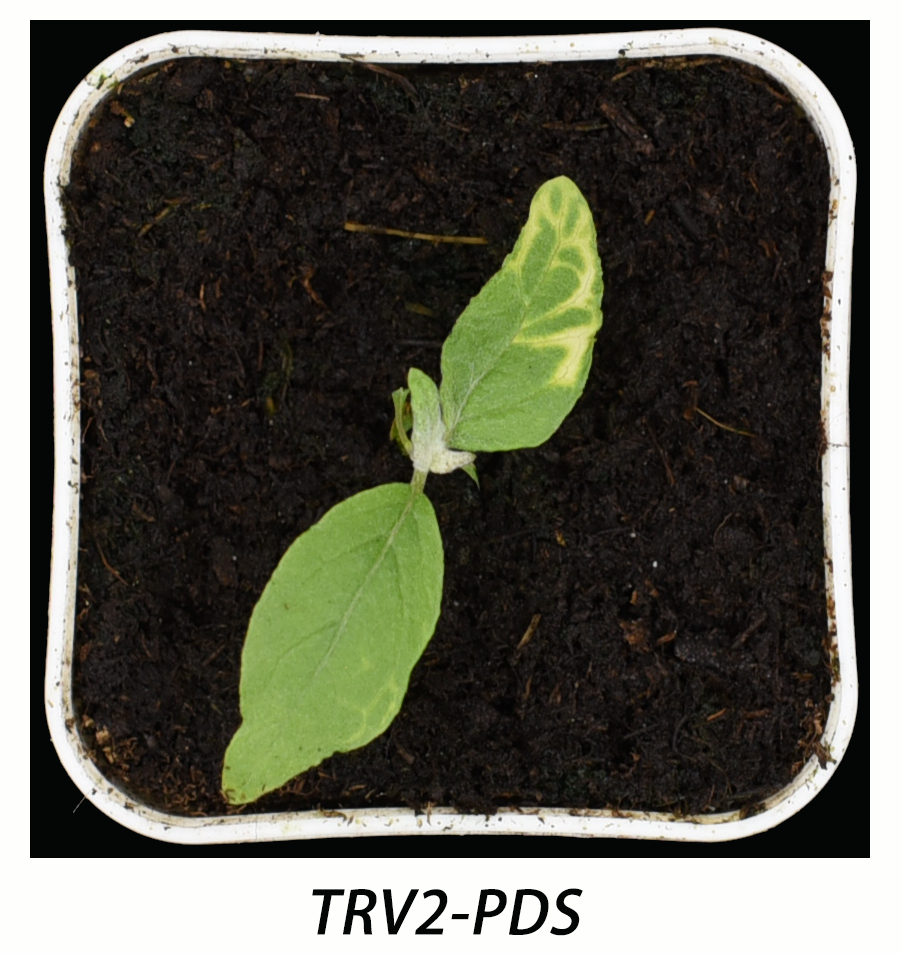

Supplement: Supplementary file 1 [file plants-10-02284-s001.zip › S3.tif]

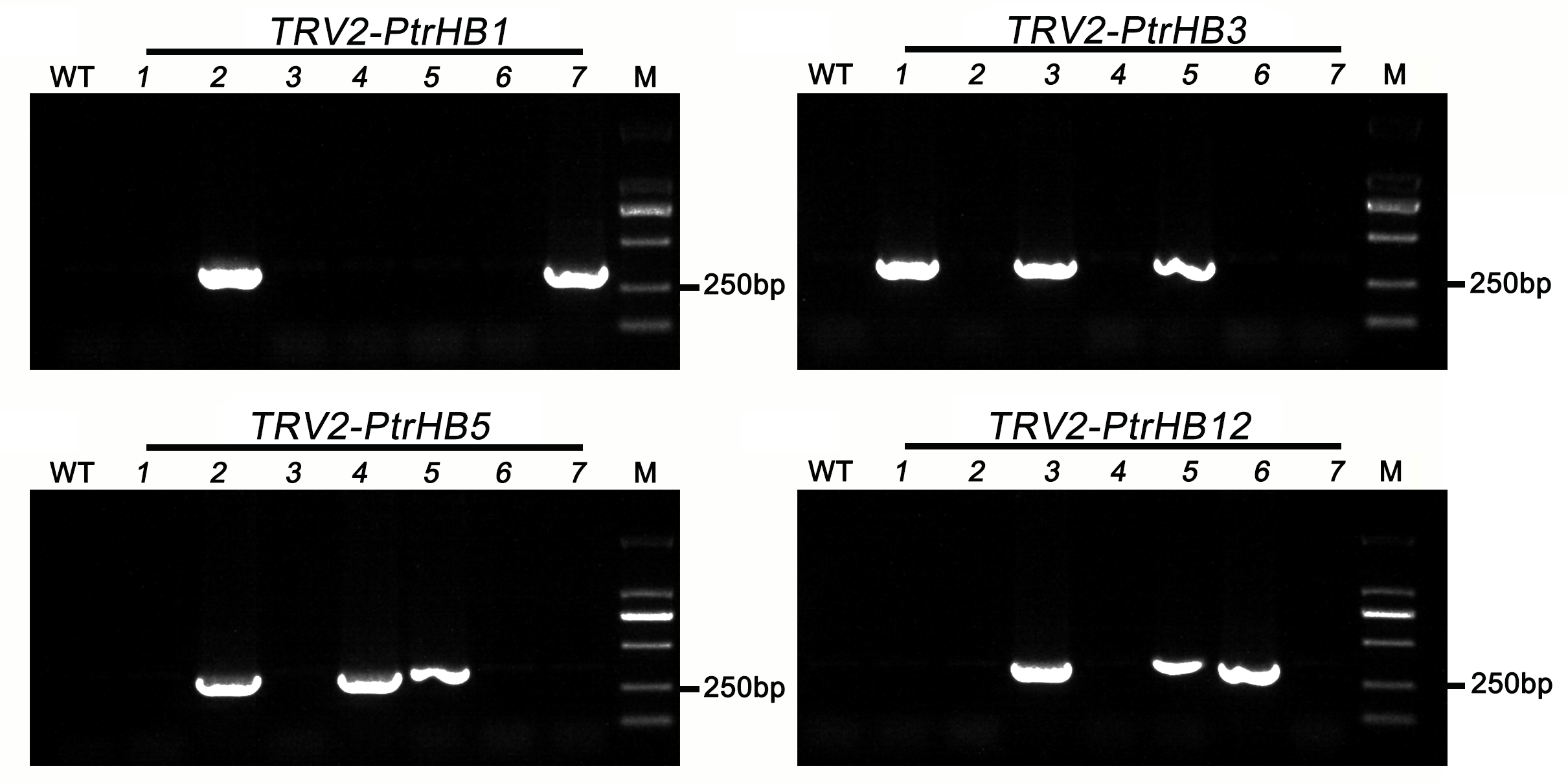

Supplement: Supplementary file 1 [file plants-10-02284-s001.zip › S4.tif]

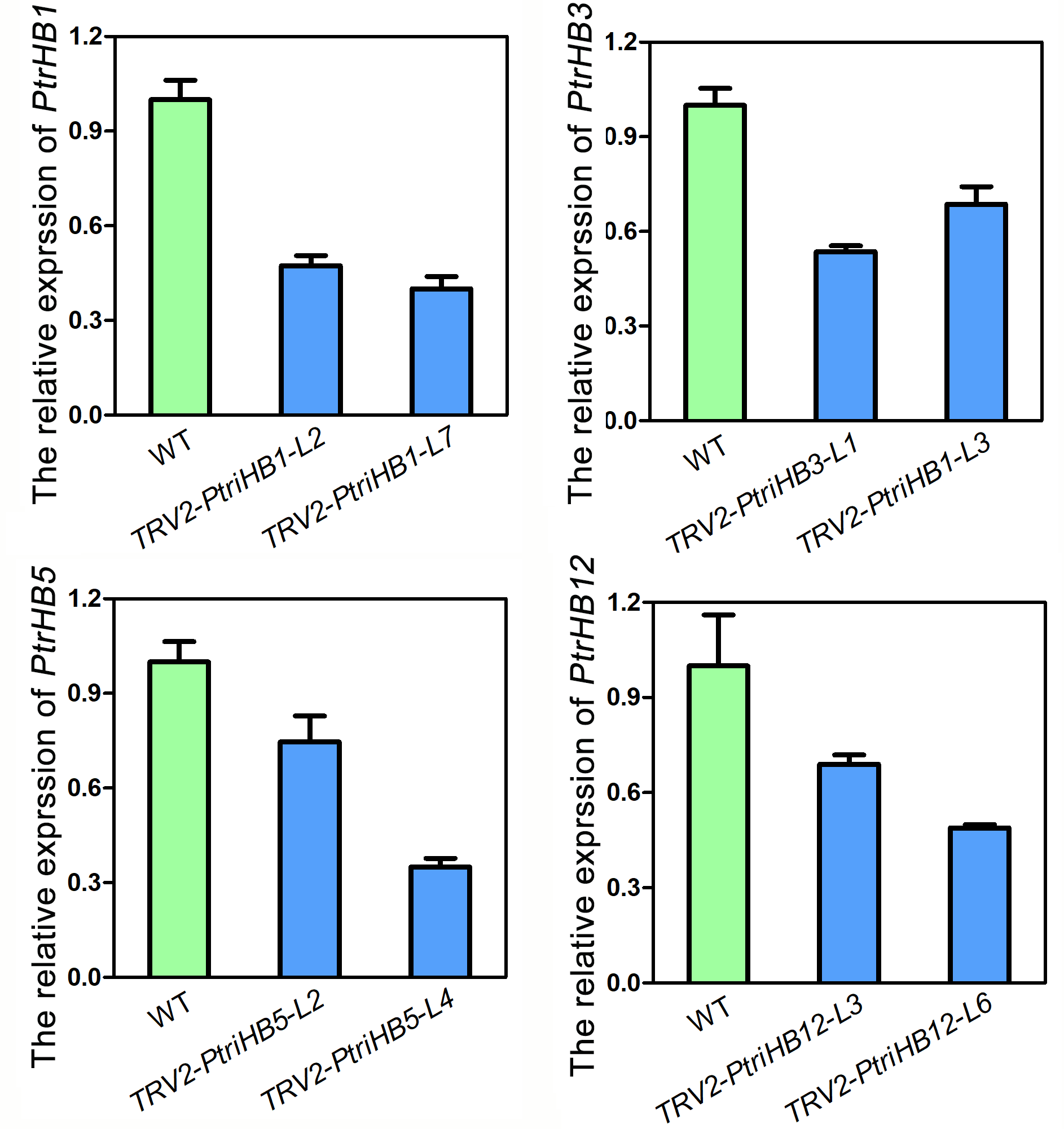

Supplement: Supplementary file 1 [file plants-10-02284-s001.zip › S5.tif]
